# Comparison of complications between laparoscopic and open gastrectomies for early gastric cancer by a nationwide propensity score-matched cohort study

**DOI:** 10.1038/s41598-023-46246-1

**Published:** 2023-11-03

**Authors:** Jeong Ho Song, Jae-Seok Min, Information Committee of the Korean Gastric Cancer Association

**Affiliations:** 1 Department of Surgery, Ajou University School of Medicine, Suwon, Republic of Korea; 2 Department of Surgery, Dongnam Institute of Radiological and Medical Sciences, Cancer Center, 40 Jwadong-gil, Jangan-eup, Gijang-gun, Busan, 46033 Republic of Korea

**Keywords:** Cancer, Gastroenterology, Medical research

## Abstract

The safety of laparoscopic gastrectomy compared with that of open surgery for the treatment of early gastric cancer (EGC) is unidentified on a national scale. We aimed to compare the morbidity between laparoscopic and open gastrectomies for pathological T1 gastric cancer based on nationwide survey data. Data of 14,076 patients who underwent gastric cancer surgery obtained from the 2019 Korean Gastric Cancer Association-led nationwide survey were used. For patients with pathological T1 gastric cancer, the clinical characteristics were compared between the laparoscopic and open gastrectomy groups. Propensity score matching (PSM) was performed to match the baseline characteristics of the groups. Among the 7765 patients with pathological T1 gastric cancer who underwent open or laparoscopic gastrectomy, 612 pairs were matched. After balancing the baseline characteristics, the laparoscopic gastrectomy group had a significantly longer operative time, less blood loss, greater number of harvested lymph nodes, shorter hospital stays, and comparable morbidity, compared with the open gastrectomy group (*P* < 0.001, *P* < 0.001, *P* < 0.001, *P* = 0.001, and *P* = 0.709, respectively). The surgical approach was not a risk factor for postoperative complication in logistic regression analysis. The PSM analysis with the 2019 Korean nationwide survey data demonstrated that laparoscopic gastrectomy showed comparable morbidity with open gastrectomy for EGC.

## Introduction

Although the mortality rate of gastric cancer has been declining, there is currently no difference in the mortality ranking among organs affected by malignant neoplasms, including the stomach, worldwide^1–4^. Gastric cancer is one of the five leading malignant neoplasms in South Korea^3,5^. Nevertheless, its incidence can be reduced by various active efforts, such as smoking cessation, alcohol abstinence, low sodium intake, body weight control, and *Helicobacter pylori* eradication^3,6–13^. If gastric cancer occurs despite efforts to prevent it, the prognosis after treatment can be favorable if gastric cancer is detected early^4^. Endoscopic examination is recommended for the early detection of gastric cancer^14^. If detected early through endoscopy, gastric cancer can be treated with endoscopic procedures that are less burdensome to patients^15,16^. However, even in cases of early gastric cancer (EGC), gastrectomy is necessary if the tumor is outside the scope of standard endoscopic treatments^17–19^.

Recently, the incidence of EGC has increased, especially in South Korea. According to the data from the Korean Gastric Cancer Association (KGCA)-led nationwide survey on surgically treated gastric cancers, the incidence rate of EGC increased from approximately 58% in 2009 to approximately 64% in 2019, and stage I gastric cancer cases accounted for approximately 66% in 2019^20^. The proportion of laparoscopic surgery in Korea was only 6.6% in 2004; it rapidly increased to 64.9% in 2019. In cases of early diagnosis, laparoscopic gastrectomy is recommended for faster postoperative recovery^20–22^. In previous studies, short-term postoperative outcomes, such as first flatus, hospital stay, and postoperative pain, were better after laparoscopic gastrectomy than after open surgery^21,23–25^. Most previous studies have reported that the morbidity rate is lower after laparoscopy than after open gastrectomy for patients with EGC^21,23–26^. However, other previous studies have not shown significantly better postoperative outcomes, including complications, after laparoscopic gastrectomy^27–29^. Moreover, there remains a lack of studies that compare the complications of the two surgical approaches based on nationwide data. In this study, we aimed to reconfirm the relationship between complications and the surgical approaches (laparoscopy vs. open) for gastrectomy in patients with EGC using the 2019 KGCA-led nationwide survey data.

## Methods

### Patients

**Figure 1:**
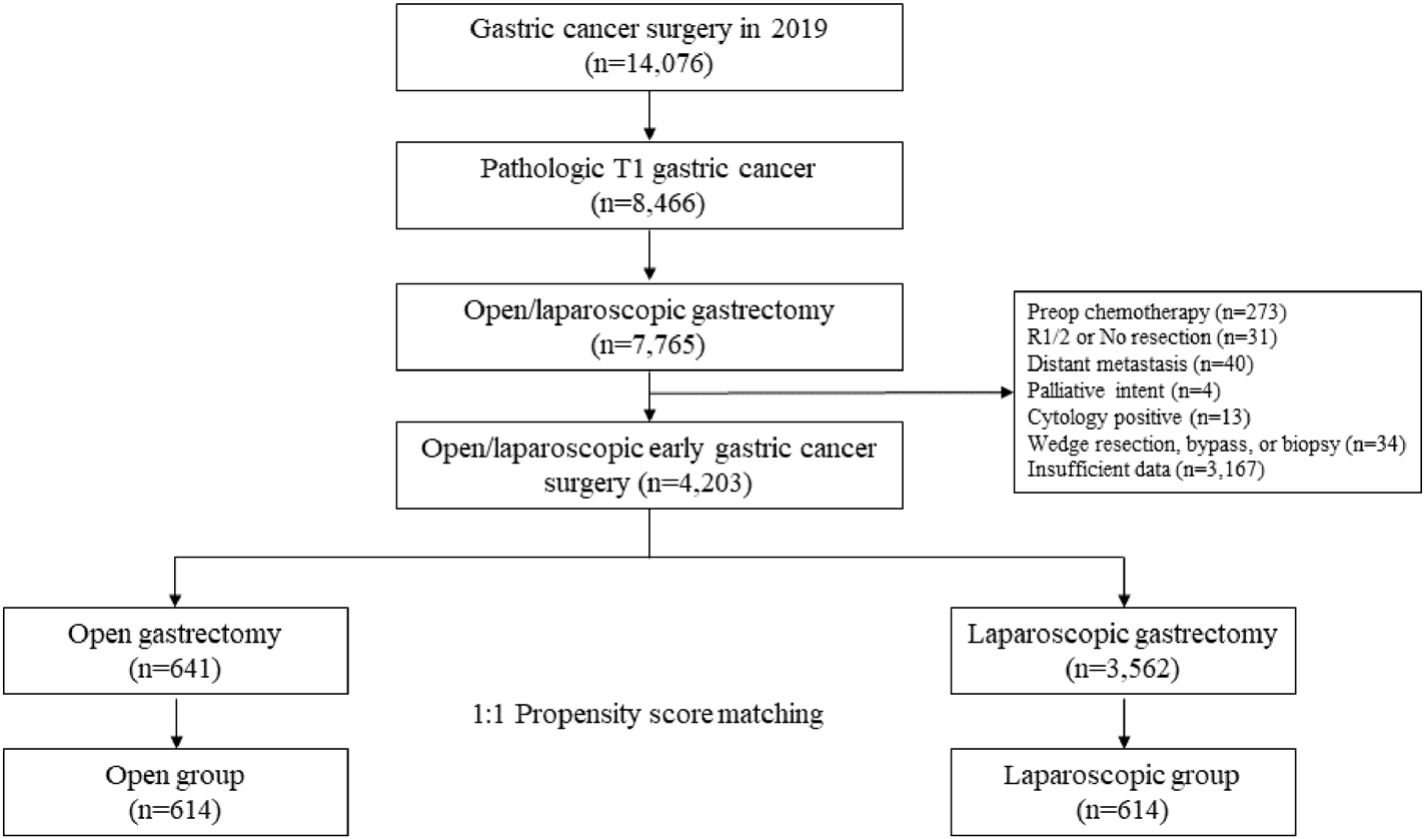
Study flowchart.

Data of 14,076 patients who underwent gastric cancer surgery obtained from the 2019 Korean nationwide survey were reviewed. Patients who underwent open or laparoscopic gastrectomy for pathological EGC (T1) were included in this study. Patients who met any of the following criteria were excluded from this study: receiving preoperative chemotherapy; undergoing non-curative resection including R1 or R2 resection; with no resection; with distant metastasis; undergoing palliative surgery; with positive cytology; undergoing wedge resection, bypass, or biopsy; and with insufficient data (Fig. 1). Insufficient data included clinicopathological features, such as American Society of Anesthesiologists (ASA) physical status classification and previous abdominal surgery, perioperative outcomes (including resection extent, operative time, blood loss, complication, and Clavien–Dindo grade), or patients’ pathological features (such as tumor size, Lauren’s classification, and lymphovascular invasion). This study was approved by the Institutional Review Boards of the Dongnam Institute of Radiological and Medical Sciences and Ajou University Hospital (D-2302-002-002 and AJOUIRB-EX-2022-551, respectively), which waived the requirement for written informed consents from the patients owing to the retrospective nature of the study. All work involving patient data was performed in accordance with the Declaration of Helsinki.

### Data

The data acquisition procedure has been described in a previous study^20^. In 2019, the Information Committee of the KGCA requested representatives from all registered institutions to collect data on gastric cancer surgery. Representative data were submitted according to the case report form of December 2020. After detailed reviewing and revisions between the Information Committee of the KGCA and each representative, the final data collection was completed in February 2021. The Information Committee of the KGCA approved the protocol of this study and also allowed us to use the 2019 KGCA-led nationwide survey data of 14,076 patients who underwent gastric cancer surgery.

The surgical procedure of gastrectomy for gastric cancer was based on the treatment guidelines for gastric cancer in Korea and Japan^30–32^. Total omentectomy was usually performed for T3 or deeper tumors or according to the surgeons’ preference. The choice of gastric resection type (distal, total, proximal, or pylorus-preserving gastrectomy) depended on the location of the tumor. Patients with EGC without suspected lymph node metastasis underwent D1 + lymphadenectomy, while D2 lymphadenectomy was performed for advanced cases or cases with suspected lymph node metastasis. Distal gastrectomy was followed by reconstruction methods such as gastroduodenostomy, gastrojejunostomy with/without braun anastomosis, Roux-en Y gastrojejunostomy, or uncut Roux-en Y gastrojejunostomy. Total gastrectomy involved Roux-en Y esophagojejunostomy or jejunal interposition, and proximal gastrectomy used double tract reconstruction or esophagogastrostomy. Gastrogastrostomy was the reconstruction method for pylorus-preserving gastrectomy. All reconstruction methods were determined by the surgeon. Overall, similar surgical procedures were applied to open and laparoscopic surgeries.

The survey data consisted of 54 items, including demographic, surgical, pathological, and perioperative characteristics. Pathological tumor stage was defined according to the 8th edition of the American Joint Committee on Cancer staging system^33^. Tumors were histologically classified as differentiated or undifferentiated according to the Japanese Classification of Gastric Carcinoma 15th edition^34^. The differentiated type consisted of papillary carcinoma or well-/moderately differentiated tubular adenocarcinoma. The undifferentiated types included poorly differentiated tubular adenocarcinoma, signet ring cell carcinoma, and mucinous carcinoma. Postoperative morbidity was defined as any complication occurring within 30 days of surgery. The severity of morbidity was classified using the Clavien–Dindo classification, and complications of grade III or higher were defined as major complications^35^.

### Statistical analyses

The propensity score matching (PSM) method was used to reduce selection bias. The variables for propensity scores included age, sex, body mass index (BMI), ASA classification, previous abdominal surgery, extent of resection, combined resection, extent of lymph node dissection, tumor size, histology, Lauren classification, depth of invasion, lymph node metastasis, pathological stage, lymphovascular invasion, and perineural invasion. Surgical approaches were matched with a caliper width of 0.1, and a 1:1 nearest-neighbor strategy without replacement was performed using the MatchIt package in R software. The standardized mean difference (SMD) was used to estimate the balance of the covariates. An absolute SMD value < 0.1 was considered a small imbalance, and the SMDs of all clinical variables were reduced to < 0.1 after matching.

The PSM identified 612 pairs of patients who underwent open or laparoscopic gastrectomy, and their perioperative outcomes were compared using the McNemar test (categorical variables) or paired T-test (continuous variables). Differences were considered statistically significant at *P* values of < 0.05. Logistic regression analysis was performed to identify the independent risk factors for morbidity and major complications. Variables with a *P* value < 0.05 in univariate analysis and surgical approach were included in the multivariate analysis. Statistical analyses were performed using IBM SPSS software for Windows (version 25.0; IBM Corp., Armonk, New York, USA) and R software (version 3.3.3; R Foundation for Statistical Computing, Vienna, Austria).

## Results

### Patient characteristics

Among the 7765 patients with pathological T1 gastric cancer who underwent open or laparoscopic gastrectomy, 3562 were excluded because of preoperative chemotherapy (N = 273); non-curative resection or no resection (N = 31); distant metastasis (N = 40); palliative intent surgery (N = 4); positive cytology (N = 13); wedge resection, bypass, or biopsy only (N = 34); and insufficient data (N = 3167). Finally, 4203 patients were included in this study, with 3562 and 641 in the laparoscopic gastrectomy and open gastrectomy groups, respectively. After PSM, 612 patient pairs were identified in both groups (Fig. 1).

The clinical, surgical, and pathological characteristics of the open and laparoscopic gastrectomy groups before and after PSM are presented in Table 1. Among all patients, the laparoscopic gastrectomy group was characterized by young age (*P* = 0.001), female sex (*P* = 0.017), low ASA score (*P* < 0.001), small tumors (*P* < 0.001), distal gastrectomy (*P* < 0.001), less than D2 lymph node dissection (*P* < 0.001), and Lauren intestinal type (*P* < 0.001). The open gastrectomy group had a higher proportion of previous abdominal surgery (*P* < 0.001), combined resection (*P* < 0.001), lymph node metastasis (*P* < 0.001), stage IB or advanced disease (*P* < 0.001), lymphovascular invasion (*P* = 0.001), and perineural invasion (*P* < 0.001) than the laparoscopic gastrectomy group. However, all these variables were well balanced between the open and laparoscopic gastrectomy groups after PSM. Further, detailed combined resection lists were not significantly different between the groups after PSM (Supplementary Table S1).

**Table 1:** Clinical, surgical, and pathological characteristics of open and laparoscopic gastrectomy approaches before and after the propensity score matching.

	Before PSM			After PSM		
	Open (N = 641)	Laparoscopy (N = 3562)	*P* value	Open (N = 612)	Laparoscopy (N = 612)	SMD
Age, years	64.0 ± 11.2	62.3 ± 11.7	0.001	63.8 ± 11.3	63.8 ± 12.1	–0.0003
Sex			0.017			0.0508
Male	438 (68.3%)	2255 (63.3%)		411 (67.2%)	396 (64.7%)	
Female	203 (31.7%)	1307 (36.7%)		201 (32.8%)	216 (35.3%)	
BMI, kg/m^2^	24.4 ± 3.4	24.3 ± 3.3	0.643	24.5 ± 3.4	24.2 ± 3.3	–0.0815
ASA			0.003			–0.0232
1	129 (20.1%)	945 (26.5%)		126 (20.6%)	152 (24.8%)	
2	409 (63.8%)	2086 (58.6%)		387 (63.2%)	344 (56.2%)	
3	103 (16.1%)	531 (14.9%)		99 (16.2%)	116 (19.0%)	
Previous abdominal surgery			< 0.001			–0.0340
Yes	156 (24.3%)	640 (18.0%)		141 (23.0%)	149 (24.3%)	
No	485 (75.7%)	2922 (82.0%)		471 (77.0%)	463 (75.7%)	
Tumor size, cm	3.31 ± 2.05	2.67 ± 1.81	< 0.001	3.2 ± 2.0	3.2 ± 2.1	–0.0199
Histology			0.888			–0.0360
Differentiated	325 (50.7%)	1820 (51.1%)		312 (51.0%)	323 (52.8%)	
Undifferentiated	316 (49.3%)	1742 (48.9%)		300 (49.0%)	289 (47.2%)	
Resection extent			< 0.001			–0.0294
DG	425 (66.3%)	3085 (86.6%)		416 (68.0%)	488 (79.7%)	
TG	207 (32.3%)	299 (8.4%)		187 (30.6%)	77 (12.6%)	
PG	9 (1.4%)	133 (3.7%)		9 (1.5%)	23 (3.8%)	
PPG	0 (0.0%)	45 (1.3%)		0	24 (3.9%)	
Combined resection			< 0.001			0.0373
Yes	65 (10.1%)	180 (5.1%)		58 (9.5%)	53 (8.7%)	
No	576 (89.9%)	3382 (94.9%)		554 (90.5%)	559 (91.3%)	
LND			< 0.001			–0.0923
Less than D2	133 (20.7%)	2303 (64.7%)		133 (21.7%)	160 (26.1%)	
D2 or more	508 (79.3%)	1259 (35.3%)		479 (78.3%)	452 (73.9%)	
Depth of invasion			0.239			0.0263
T1a	336 (52.4%)	1960 (55.0%)		316 (51.6%)	308 (50.3%)	
T1b	305 (47.6%)	1602 (45.0%)		296 (48.4%)	304 (49.7%)	
LN metastasis			< 0.001			0.0059
N0	556 (86.7%)	3266 (91.7%)		536 (87.6%)	535 (87.4%)	
N +	85 (13.3%)	296 (8.3%)		76 (12.4%)	77 (12.6%)	
Stage			< 0.001			0. 0059
IA	556 (86.7%)	3266 (91.7%)		536 (87.6%)	535 (87.4%)	
IB or advanced	85 (13.3%)	296 (8.3%)		76 (12.4%)	77 (12.6%)	
Lauren classification			< 0.001			–0.0498
Intestinal	311 (48.5%)	1823 (51.2%)		302 (49.3%)	310 (50.7%)	
Diffused	203 (31.7%)	1318 (37.0%)		195 (31.9%)	200 (32.7%)	
Mixed	127 (19.8%)	421 (11.8%)		115 (18.8%)	102 (16.7%)	
LVI			0.001			0.0567
Yes	121 (18.9%)	494 (13.9%)		109 (17.8%)	97 (15.8%)	
No	520 (81.1%)	3068 (86.1%)		503 (82.2%)	515 (84.2%)	
PNI			< 0.001			–0.0615
Yes	48 (7.5%)	93 (2.6%)		35 (5.7%)	41 (6.7%)	
No	593 (92.5%)	3469 (97.4%)		577 (94.3%)	571 (93.3%)	

Data are shown as means ± standard deviations or numbers (proportions).

*PSM* propensity score matching; *SMD* standardized mean difference; *BMI* body mass index; *ASA* American Society of Anesthesiologists physical status classification; *DG* distal gastrectomy; *TG* total gastrectomy; *PG* proximal gastrectomy; *PPG* pylorus-preserving gastrectomy; *LND* lymph node dissection; *LN* lymph node; *LVI* lymphovascular invasion; *PNI* perineural invasion.

### Perioperative outcomes

Perioperative outcomes between the open and laparoscopic gastrectomy groups before and after PSM are shown in Table 2. In all patients, the laparoscopic gastrectomy group had a significantly longer operative time, less blood loss, greater number of harvested lymph nodes, shorter hospital stays, and less receipt of adjuvant chemotherapy compared with the open gastrectomy group (*P* = 0.005, *P* < 0.001, *P* < 0.001, *P* < 0.001, and *P* < 0.001, respectively). These perioperative outcomes were significant after PSM (*P* < 0.001 for operative time, *P* < 0.001 for blood loss, *P* < 0.001 for number of harvested lymph nodes, *P* = 0.001 for hospital stay, and *P* = 0.039 for adjuvant chemotherapy). Morbidity rate was not significantly different between groups before and after PSM (*P* = 0.721 and *P* = 0.709, respectively). No significant differences were observed between the two groups before and after PSM according to the Clavien–Dindo classification of complications (all *P* > 0.05).

**Table 2:** Perioperative outcomes of open and laparoscopic gastrectomy approaches before and after the propensity score matching.

	Before PSM			After PSM		
	Open (N = 641)	Laparoscopy (N = 3562)	*P* value	Open (N = 612)	Laparoscopy (N = 612)	*P* value
Operative time, min	173.0 ± 60.5	180.3 ± 59.3	0.005	173.0 ± 60.5	186.60 ± 61.5	< 0.001
Blood loss, mL	159.4 ± 162.7	73.4 ± 112.8	< 0.001	158.5 ± 161.9	71.8 ± 74.1	< 0.001
Number of harvested LNs	36.2 ± 15.6	39.3 ± 16.4	< 0.001	35.9 ± 15.4	40.4 ± 16.2	< 0.001
Hospital stay, days	10.2 ± 6.9	8.3 ± 6.8	< 0.001	10.3 ± 7.1	8.9 ± 7.1	0.001
Morbidity						
No	571 (89.1%)	3193 (89.6%)	0.721	546 (89.2%)	541 (88.4%)	0.709
Yes	70 (10.9%)	369 (10.4%)		66 (10.8%)	71 (11.6%)	
C–D grade						
I	1 (2.2%)	127 (3.6%)	0.074	13 (2.1%)	13 (2.1%)	> 0.999
II	40 (6.2%)	179 (5.0%)	0.203	37 (6.0%)	34 (5.6%)	0.798
IIIa	12 (1.9%)	56 (1.6%)	0.580	12 (2.0%)	12 (2.0%)	> 0.999
IIIb	7 (1.1%)	44 (1.2%)	0.760	7 (1.1%)	11 (1.8%)	0.480
IVa	6 (0.9%)	19 (0.5%)	0.222	6 (1.0%)	4 (0.7%)	0.752
V	2 (0.3%)	13 (0.4%)	0.836	1 (0.2%)	4 (0.7%)	0.371
Adjuvant chemotherapy						
Yes	58 (9.0%)	102 (2.9%)	< 0.001	48 (7.8%)	30 (4.9%)	0.039
No	583 (91.0%)	3460 (97.1%)		564 (92.2%)	582 (95.1%)	

Data are shown as means ± standard deviations or numbers (proportions).

*PSM* propensity score matching; *LNs* lymph nodes; *C–D grade* Clavien–Dindo grade.

Major complications of Clavien–Dindo grade III or higher complications occurred in 54 patients after PSM. Detailed lists of major complications are summarized in Table 3. Wound complication was notably more common in open gastrectomy than in laparoscopic surgery (*P* = 0.012). The incidences of other detailed complications were not significantly different between the two groups (all *P* > 0.05).

**Table 3:** Comparison of major complications according to surgical approach after the propensity score matching.

	Open (N = 612)	Laparoscopy (N = 612)	*P* value
Anastomosis leakage	3 (0.5%)	7 (1.1%)	0.204
Anastomosis stenosis	0	2 (0.3%)	0.157
Duodenal stump leakage	0	2 (0.3%)	0.157
Intraabdominal bleeding	2 (0.3%)	3 (0.5%)	0.654
Luminal bleeding	0	2 (0.3%)	0.157
Pancreatic fistula	0	1 (0.2%)	0.317
Intraabdominal abscess	2 (0.3%)	1 (0.2%)	0.563
Fluid collection	2 (0.3%)	1 (0.2%)	0.563
Wound problem	11 (1.8%)	2 (0.3%)	0.012
Mechanical ileus	2 (0.3%)	5 (0.8%)	0.255
Pneumonia	0	2 (0.3%)	0.157
Cerebrovascular accident	1 (0.2%)	0	0.317
Heart problem	2 (0.3%)	1 (0.2%)	0.563
Others	3 (0.5%)	3 (0.5%)	> 0.999

Data are shown as numbers (proportions).

### Risk factors for morbidity

Of the 1224 matched patients, morbidity occurred in 137 (11.2%) patients. In the univariate analysis, age, sex, previous abdominal surgery, histology, resection extent, combined resection, and Lauren classification were associated with morbidity (*P* = 0.028, *P* < 0.001, *P* = 0.042, *P* = 0.001, *P* = 0.032, *P* = 0.017, and *P* = 0.005, respectively) (Table 4). Other variables, including body mass index, ASA score, tumor size, surgical approach, extent of lymph node dissection, depth of tumor invasion, lymph node metastasis, pathological stage, lymphovascular invasion, and perineural invasion, were not significantly different between the two groups (all *P* > 0.05). Multivariate analysis identified male sex (odds ratio [OR], 2.232; *P* = 0.001), previous abdominal surgery (OR, 1.772; *P* = 0.006), total gastrectomy (OR, 1.794; *P* = 0.005), and combined resection (OR, 1.929; *P* = 0.016) as independent risk factors for morbidity. The surgical approach was not an independent risk factor for morbidity (*P* = 0.300).

**Table 4:** Risk factors for morbidity after the propensity score matching.

	Univariate analysis			Multivariate analysis		
	Morbidity (–) (N = 1087)	Morbidity ( +) (N = 137)	*P* value	Exp(B)	95% CI	*P* value
Age, years	63.6 ± 11.8	65.7 ± 10.3	0.028	1.008	0.991–1.026	0.**359**
Sex						
Female	390 (35.9%)	27 (19.7%)	< 0.001	1	1.409–3.535	0.001
Male	697 (64.1%)	110 (80.3%)		2.232		
BMI, kg/m^2^	24.3 ± 3.4	24.6 ± 3.2	0.328			
ASA						
1	252 (23.2%)	26 (19.0%)				
2	653 (60.1%)	78 (56.9%)	0.087			
3	182 (16.7%)	33 (24.1%)				
Previous abdominal surgery						
No	839 (77.2%)	95 (69.3%)	0.042	1	1.181–2.659	0.006
Yes	248 (22.8%)	42 (30.7%)		1.772		
Tumor size, cm	3.2 ± 2.0	3.1 ± 1.9	0.562			
Histology						
Differentiated	546 (50.2%)	89 (65.0%)	0.001	1		0.557
Undifferentiated	541 (49.8%)	48 (35.0%)		0.851	0.497–1.458	
Surgical approach						
Open	546 (50.2%)	66 (48.2%)	0.650	1		0.300
Laparoscopy	541 (49.8%)	71 (51.8%)		1.221	0.837–1.780	
Resection extent						
DG	817 (75.2%)	87 (63.5%)		1		
TG	222 (20.4%)	42 (30.7%)		1.794	1.196–2.690	0.005
PG	27 (2.5%)	5 (3.6%)		1.872	0.686–5.113	0.221
PPG	21 (1.9%)	3 (2.2%)	0.032	1.445	0.412–5.070	0.566
Combined resection			0.017			
No	996 (91.6%)	117 (85.4%)		1		0.016
Yes	91 (8.4%)	20 (14.6%)		1.929	1.129–3.296	
LND						
Less than D2	256 (23.6%)	37 (27.0%)	0.372			
D2 or more	831 (76.4%)	100 (73.0%)				
Depth of invasion						
T1a	554 (51.0%)	70 (51.1%)	0.977			
T1b	533 (49.0%)	67 (48.9%)				
LN metastasis						
N0	946 (87.0%)	125 (91.2%)	0.160			
N +	141 (13.0%)	12 (8.8%)				
Stage						
IA	946 (87.0%)	125 (91.2%)	0.160			
IB or advanced	141 (13.0%)	12 (8.8%)				
Lauren classification						
Intestinal	527 (48.5%)	85 (62.0%)		1		
Diffused	366 (33.7%)	29 (21.2%)	0.005	0.709	0.380–1.324	0.281
Mixed	194 (17.8%)	23 (16.8%)		0.954	0.539–1.689	0.873
LVI						
Yes	184 (16.9%)	22 (16.1%)	0.798			
No	903 (83.1%)	115 (83.9%)				
PNI						
Yes	67 (6.2%)	9 (6.6%)	0.853			
No	1020 (93.8%)	128 (93.4%)				

Data are shown as means ± standard deviations or numbers (proportions).

Significant values are in [bold].

*CI* confidence interval; *BMI* body mass index; *ASA* American Society of Anesthesiologists physical status classification; *DG* distal gastrectomy; *TG* total gastrectomy; *PG* proximal gastrectomy; *PPG* pylorus-preserving gastrectomy; *LND* lymph node dissection; *LN* lymph node; *LVI* lymphovascular invasion; *PNI* perineural invasion.

Univariate and multivariate analyses for major complications are demonstrated in Table 5. Univariate analysis identified age, sex, histology, extent of tumor resection, and combined resection as significant predictors of major complications (*P* = 0.020, *P* = 0.001, *P* = 0.002, *P* = 0.028, and *P* = 0.001, respectively) (Table 5). The surgical approach was not associated with major complications (*P* = 0.578). In multivariate analysis, male sex (OR, 3.219; *P* = 0.005), total gastrectomy (OR, 1.940; *P* = 0.033), and combined resection (OR, 3.034; *P* = 0.002) were independent risk factors for major complications. The surgical approach was not an independent risk factor for major complications (*P* = 0.441).

**Table 5:** Risk factors for major complications after the propensity score matching.

	Univariate analysis			Multivariate analysis		
	Major complications (–) (N = 1170)	Major complications ( +) (N = 54)	*P* value	Exp(B)	95% CI	*P* value
Age, years	63.7 ± 11.7	67.5 ± 10.2	0.020	1.026	0.998–1.056	0.074
Sex						
Female	410 (35.0%)	7 (13.0%)	0.001	1		0.005
Male	760 (65.0%)	47 (87.0%)		3.219	1.413–7.336	
BMI, kg/m^2^	24.3 ± 3.4	24.5 ± 3.6	0.620			
ASA						
1	266 (22.7%)	12 (22.2%)	0.648			
2	701 (59.9%)	30 (55.6%)				
3	203 (17.4%)	12 (22.2%)				
Previous abdominal surgery						
Yes	278 (23.8%)	12 (22.2%)	0.795			
No	892 (76.2%)	42 (77.8%)				
Tumor size, cm	3.2 ± 2.2	3.6 ± 2.6	0.199			
Histology						
Undifferentiated	574 (49.1%)	15 (27.8%)	0.002	1		0.086
Differentiated	596 (50.9%)	39 (72.2%)		1.744	0.924–3.292	
Surgical approach						
Open	587 (50.2%)	25 (46.3%)	0.578	1		0.441
Laparoscopy	583 (49.8%)	29 (53.7%)		1.261	0.699–2.275	
Resection extent						
DG	872 (74.5%)	32 (59.3%)		1		
TG	246 (21.0%)	18 (33.3%)	0.028	1.940	1.055–3.566	0.033
PG	31 (3.1%)	1 (1.9%)		0.869	0.112–6.736	0.893
PPG	21 (1.2%)	3 (5.6%)		4.836	1.301–17.979	0.019
Combined resection			0.001			
No	1071 (91.5%)	42 (77.8%)		1		0.002
Yes	99 (8.5%)	12 (22.2%)		3.034	1.507–6.107	
LND						
Less than D2	279 (23.8%)	14 (25.9%)	0.726			
D2 or more	891 (76.2%)	40 (74.1%)				
Depth of invasion						
T1a	598 (51.1%)	26 (48.1%)	0.670			
T1b	572 (48.9%)	28 (51.9%)				
LN metastasis						
N0	1021 (87.3%)	50 (92.6%)	0.247			
N +	149 (12.7%)	4 (7.4%)				
Stage						
IA	1021 (87.3%)	50 (92.6%)	0.247			
IB or advanced	149 (12.7%)	4 (7.4%)				
Lauren classification						
Intestinal	577 (49.3%)	35 (64.8%)				
Diffused	385 (32.9%)	10 (18.5%)	0.054			
Mixed	208 (18.0%)	9 (16.7%)				
LVI						
Yes	200 (17.1%)	6 (11.1%)	0.251			
No	970 (82.9%)	48 (88.9%)				
PNI						
Yes	71 (6.1%)	5 (9.3%)	0.342			
No	1099 (93.9%)	49 (90.7%)				

Clavien–Dindo classification grade III or higher are defined as major complications.

Data are shown as means ± standard deviations or numbers (proportions).

*CI* confidence interval; *BMI* body mass index; *ASA* American Society of Anesthesiologists physical status classification; *DG* distal gastrectomy; *TG* total gastrectomy; *PG* proximal gastrectomy; *PPG* pylorus-preserving gastrectomy; *LND* lymph node dissection; *LN* lymph node; *LVI* lymphovascular invasion; *PNI* perineural invasion.

## Discussion

In our study, we compared the complications between laparoscopic and open gastrectomies in EGC by performing PSM analysis to minimize selection bias based on the KGCA-led nationwide survey data. There was no significant difference in postoperative morbidity, major complications, and mortality between laparoscopic and open gastrectomies for EGC. Among all complications, wound complications were the only ones associated with a higher incidence after laparoscopic surgery than after open gastrectomy.

According to the Korean Practice Guidelines for Gastric Cancer 2022, laparoscopic distal gastrectomy is recommended for clinical stage I gastric cancer^17^. The Korean multicenter randomized controlled trial (RCT) (KLASS-01) reported that laparoscopic distal gastrectomy for clinical stage I gastric cancer was safe and showed a lower incidence of wound complications than open distal gastrectomy^21^. In contrast, a systematic review and meta-analysis reported that the distributions of complication grades based on the Clavien–Dindo classification were not different between laparoscopic and open gastrectomies^36^; in this meta-analysis, laparoscopic gastrectomy had lower rates of wound complications and intra-abdominal fluid collection compared with open gastrectomy. Other complications did not differ significantly between the two approaches. In addition, laparoscopic gastrectomy was associated with a longer operation time and shorter postoperative hospital stay than open gastrectomy, which is consistent with the findings of our study. A Western multicenter randomized trial (LOGICA trial) reported that postoperative complications did not differ between laparoscopic and open gastrectomies^27^. Similar to the present study, the LOGICA trial included patients who underwent total and distal gastrectomies. An RCT compared the safety of laparoscopic total gastrectomy (LTG) for clinical stage I gastric cancer with that of open total gastrectomy (OTG). A total of 214 patients (105 in the LTG group and 109 in the OTG group) were analyzed for morbidity and mortality. Postoperative morbidity and mortality rates were not significantly different between the LTG and OTG groups. Another feasibility study (KLASS-03) showed acceptable morbidity and mortality compared with those of a previous study on OTG^37^. Aforementioned several pivotal RCTs were conducted by selected surgeons who performed many laparoscopic surgeries, especially in South Korea. In this study, where we collected nationwide data on gastric cancer surgeries, regardless of the number of gastrectomies performed by a surgeon, the postoperative complications of laparoscopic gastrectomy were comparable to those of open gastrectomy. Therefore, this study can prove the efficacy of laparoscopic gastrectomy including total gastrectomy regardless of the surgeon’s experience of laparoscopic surgery.

Several previous studies have suggested the risk factors for complications after gastric cancer surgery, and the KLASS-01 for EGC described the open approach and number of patient comorbidities as independent risk factors for postoperative complications^21^. In this study, where we performed PSM analysis based on the KGCA-led nationwide survey data, the operative approach for gastrectomy was not a risk factor for postoperative complications. Surgical experience overcoming learning curve was revealed to be a factor influencing complications after laparoscopic distal or total gastrectomy^38–40^. Therefore, laparoscopic surgery for gastrectomy is considered a more demanding procedure than open surgery in terms of technique and safety^36^. This study documented several risk factors for morbidity and major complications such as female sex, total gastrectomy, and combined resection, in accordance with previous studies^41–43^.

This study had some limitations. First, as this was a retrospective multicenter study, some data were missing. Despite nationwide data, the number of patients included in the analysis was limited owing to a large amount of missing data. Furthermore, detailed analyses of the specific types of complications were limited. Second, the detailed number of gastrectomies performed by a surgeon was missing in this nationwide data. Therefore, it was not possible to analyze the relationship between surgeons’ experience and postoperative complications. Third, several patients underwent D2 lymph node dissection (open gastrectomy group, 78.3%; laparoscopic gastrectomy group, 73.9%). According to the Korean Practice Guidelines for Gastric Cancer 2022, D1 + lymph node dissection can be performed in patients with EGC with negative lymph node metastasis^17^. Nevertheless, to the best of our knowledge, this is the first study to compare and analyze complications using PSM by collecting large-scale patient data from the KGCA-led nationwide survey.

In conclusion, laparoscopic gastrectomy showed similar complication rates to open gastrectomy in this study, where we used the PSM method based on the KGCA-led nationwide survey data. Surgical approach was not a risk factor for complications after gastrectomy for EGC.

### Supplementary Information

Supplementary Table S1.

## Data Availability

The datasets generated during and/or analyzed during the current study are not publicly available, however are available from the corresponding author on reasonable request after approval of Information Committee of the Korean Gastric Cancer Association.
