# Supplementary material for: Comparison of complications between laparoscopic and open gastrectomies for early gastric cancer by a nationwide propensity score-matched cohort study
Source: Sci Rep. 2023 Nov 3;13:18970. doi: 10.1038/s41598-023-46246-1 (PMC10624863; doi:10.1038/s41598-023-46246-1)
Supplement: Supplementary file 1 — Supplementary Table S1. [file 41598_2023_46246_MOESM1_ESM.pdf]

Supplementary Table S1. Comparison of detailed combined resection lists according to surgical approach after the propensity score matching

	Open (N=612)	Laparoscopy (N=612)	P-value
Spleen	5 (0.8%)	1 (0.2%)	0.218
Pancreas	2 (0.3%)	0	0.500
Transverse colon	3 (0.5%)	1 (0.2%)	0.624
Gallbladder	42 (6.9%)	43 (7.0%)	0.910
Adrenal gland	0	0	N/A
Liver	5 (0.8%)	11 (0.2%)	0.218
Diaphragm	0	0	N/A
Mesocolon	2 (0.3%)	0	0.500
Abdominal wall	2 (0.3%)	1 (0.2%)	>0.999
Unknown	2 (0.3%)	6 (1.0%)	0.287

Data are shown as numbers (proportions).

N/A=Not applicable
